# Influence of total sugar intake on metabolic blood markers at 8 years of age in the Childhood Obesity Project

**DOI:** 10.1007/s00394-020-02229-w

**Published:** 2020-05-06

**Authors:** Nicole Aumueller, Dariusz Gruszfeld, Kinga Gradowska, Joaquín Escribano, Natalia Ferré, Françoise Martin, Pascale Poncelet, Elvira Verduci, Alice ReDionigi, Berthold Koletzko, Veit Grote

**Affiliations:** 1 Division of Metabolic and Nutritional Medicine, Department of Paediatrics, Dr. von Hauner Children’s Hospital, LMU-Ludwig-Maximilians-Universität Munich, Lindwurmstr. 4, 80337 Munich, Germany; 2 Neonatal Intensive Care Unit, Children’s Memorial Health Institute, Warsaw, Poland; 3 Hospital Universitari Sant Joan de Reus, Reus, Spain; 4 Paediatrics Research Unit, Universitat Rovira I Virgili, IISPV, Reus, Spain; 5 CHC St. Vincent, Liège-Rocourt, Belgium; 6 Queen Fabiola Children’s University Hospital, Brussels, Belgium; 7 Department of Peadiatrics, San Paolo Hospital, University of Milan, Milan, Italy

**Keywords:** Sugar intake, Children, Blood lipids, Blood sugars, HDL

## Abstract

**Purpose:**

We aimed to characterize the association of dietary sugar intake with blood lipids and glucose-related markers in childhood.

**Methods:**

Data from the multicentric European Childhood Obesity Project Trial were used. Three-day weighed dietary records were obtained at 8 years of age along with serum concentrations of triglycerides, total cholesterol, low-density lipoprotein cholesterol, high-density lipoprotein cholesterol (HDL-C), glucose, and insulin. Total sugar intake comprised all mono- and disaccharides; different sugar sources were defined. Linear regression models were applied to investigate the cross-sectional association of total sugar intake with blood lipids and glucose-related markers with adjustment for total energy intake using the residual method.

**Results:**

Data were available for 325 children. Children consumed on average 332 kcal (SD 110) and 21% (SD 6) of energy from total sugar. In an energy-adjusted model, an increase of 100 kcal from total sugar per day was significantly associated with a *z* score HDL-C decrease (− 0.14; 95% CI − 0.01, − 0.27; *p* value = 0.031). Concerning different food groups of total sugar intake, 100 kcal total sugar from sweetened beverages was negatively associated with *z* score HDL-C (− 1.67; 95% CI − 0.42, − 2.91; *p* value = 0.009), while total sugar from milk products was positively related to *z* score HDL-C (1.38, 95% CI 0.03, 2.72; *p* value = 0.045). None of the other blood lipids or glucose-related markers showed a significant relationship with total sugar intake.

**Conclusion:**

Increasing dietary total sugar intake in children, especially from sweetened beverages, was associated with unfavorable effects on HDL-C, which might increase the long-term risk for dyslipidemia and cardiovascular disease.

**Clinical trial registry:**

ClinicalTrials.gov Identifier: NCT00338689; Registered: June 19, 2006. URL: https://clinicaltrials.gov/ct2/show/NCT00338689?term=NCT00338689&rank=1.

**Electronic supplementary material:**

The online version of this article (10.1007/s00394-020-02229-w) contains supplementary material, which is available to authorized users.

## Introduction

About 41 million deaths worldwide were caused by non-communicable diseases (NCDs) in 2016. The majority of those deaths are attributed to four main NCDs: cardiovascular disease (CVD) (44% of all NCD deaths), cancer (22%), chronic respiratory disease (9%), and diabetes (4%) [1]. Concerning CVDs, dyslipidemia and elevated blood pressure are major risk factors exacerbating the development of atherosclerotic plaques [2, 3]. Already in childhood, the prevalence of dyslipidemia and high blood pressure is increasing [4] and these risk factors during childhood predict higher risks of hypertension, dyslipidemia, and CVD in adulthood [5–7]. Elevated fasting glucose is another risk factor for CVDs [8], and hyperglycaemia in childhood is known to enhance the likelihood of developing insulin resistance and diabetes [9, 10]. Because of the early onset of NCDs and the propagation of its development by unfavorable metabolic risk factors in childhood, effective interventions need to be identified and implemented.

In addition to tobacco use, air pollution, physical inactivity, and harmful use of alcohol, a poor diet quality has been identified as a major risk factor for NCDs [1, 11]. A diet with high sugar intake has been suggested to lead to a poor diet quality [12]. Although sugar intake seems no longer to increase and even partly to decrease [13, 14], high sugar intake is still highly prevalent in the last few decades both in adults and children and lies above the World Health Organization (WHO) recommendation of free sugar intake beyond 10% of energy percentage [12, 15]. Previous studies indicated that high intakes of sugar and particularly added sugar (sugars added to foods or beverages during food production, processing, or preparation) [12], and sugar-sweetened beverages (SSB), are associated with unfavorable blood lipid and glucose concentrations [16–25]. This comprises high triglyceride (TG) and total cholesterol (TC) as well as low-density lipoprotein cholesterol (LDL-C), and low high-density lipoprotein cholesterol (HDL-C) concentrations [16–22], along with concentrations of glucose and insulin, and high homeostasis model assessment (HOMA) levels [23–25]. Results might differ between type of sugar assessed (e.g. total sugar, added sugar, or specific sugars such as fructose) and food sources of sugar intake (e.g. SSBs, liquid or solid) [16, 26, 27]. Nevertheless, results from published studies in children are still inconclusive [15, 26, 28] and the evidence for total sugar intake in children and its relation to NCD risk markers measured in the blood is limited. Therefore, we aimed to investigate the association of total sugar intake as well as major food sources of total sugar with blood markers related to different health outcomes, such as blood lipids and glucose-related markers, in 8-year-old children.

## Methods

### Study design

Data were used from the CHildhood Obesity Project (CHOP), a study that was originally set up as a double-blind randomized nutritional intervention trial during the first year of life in five European countries (Belgium, Germany, Italy, Poland, Spain). Details are described elsewhere [29]. Briefly, the aim of CHOP was to explore the effects of lower and higher protein supply with infant formula on growth and subsequent obesity risk. The study also included a reference group of breastfed children. Between October 2002 and July 2004, 1678 healthy infants born after uncomplicated singleton pregnancies were enrolled. Some children (*N* = 589) were followed until 8 years of age. As investigated blood markers are time variable, and exposure and outcome were only measured at the same time at 8 years in children of the original intervention trial. Therefore, a cross-sectional design was chosen for the current investigation. Written informed consent was obtained from the parents at enrollment. Additionally, children gave their assent at 8 years of age. The trial was approved by the ethics committees and follows the Declaration of Helsinki (Registration number: NCT00338689).

### Nutritional assessment

Dietary intake was assessed at 8 years of age using weighed dietary records on three consecutive days, including one weekend day. Foods and leftovers were weighed by parents, caregivers, or the children themselves following detailed instruction. Outside home, families had the opportunity to compare the amount of consumed food with standardized and weighed portion sizes mapped in an especially designed alternative dietary record. Returned dietary records were validated by trained dieticians, entered into the database, and checked for quality by standard operating procedures, which are explained more in detail elsewhere [30]. Briefly, trained dietitians checked the records, and in case of implausible or missing information, parents were asked to complete or clarify the records by phone. Additionally, each single day was checked for accuracy by a standardized score and data entry was monitored by a food record and nutritionist [30]. Nutrient intakes were calculated using the German food composition database BLS 3.01 (Bundeslebensmittelschlüssel Version 3.01). The database was chosen as it was at the time of study beginning the most comprehensive and available one in all study countries. Items not listed in the BLS, including country-specific foods, were added to the food database by study dieticians on the basis of nutrient content from the food manufacturer or other nutritional databases. Total sugar content in each food product was calculated as a composition of mono- and disaccharides, including all natural and added sugars [31].

### Blood markers

At the age of 8 years, venous blood was mainly drawn in the morning after at least 6 h fasting by trained study nurses applying same standard operating procedures. In Germany, however, blood was drawn in the afternoon without 6-h fasting in some cases. Blood samples were stored at − 70 °C and transported on dry ice to the central laboratory (Children’s Memorial Health Institute, Warsaw, Poland). Insulin was measured with an immunoradiometric assay (DiaSource, Nivelles, Belgium). All other markers, LDL-C, HDL-C, TC, TG, and glucose, were measured in the laboratories of the local study centers according to the local hospital routines including measures of quality control. In all centers, enzymatic methods were used. TG/HDL-C-ratio and HOMA index [HOMA index = (insulin (µu/ml) × glucose (mg/dl))/405] were calculated. As methods could not be standardized across centers, it has to be assumed that differences between study centers are at least partially due to methodological differences. This has been taken into account by calculating *z* scores for the individual centers.

### Covariates

Most potential risk factors or confounders were assessed at baseline by questionnaires. Maternal pre-pregnancy weight (kg), smoking, and alcohol drinking during pregnancy (yes/no) were reported by the mother; maternal height (cm) was measured at the study site. Parental education was assessed in accordance with the International Standard Classification of Education and categorized into low, middle, and high [32]. Anthropometric measures of the children were assessed by trained study nurses, following standard operating procedures [33] at 8 years of age. Body mass index [BMI = weight (kg)/height (m)^2^] was calculated and standardized to the WHO reference population to compute age- and sex-specific BMI z scores (zBMI) [34]. Physical activity was measured with the SenseWear Armband 2 (Body Media Inc., Pittsburgh, USA) on at least three consecutive days for at least 20 h per day, as described in detail elsewhere [35]. Physical active was classified by average Metabolic Equivalents of Task > 1.5. Misreporting of TEI was classified based on the comparison to the child’s individual energy expenditure, calculated from height and weight, as described in detail by Gomes et al. [36]. Based on this comparison, children were afterwards classified into either a correct reporting group, under-reporting group or over-reporting group (correct reporting: 70%, under-reporting: 27%, over-reporting: 3%).

### Statistical analysis

Nutritional data were validated during data introduction by trained dietitians and checked for plausibility by comparing nutritional intake to individual weight-, height-, and age-dependent energy intakes and needs for vitamins and minerals, which is reported more in detail elsewhere [30]. Observations with values higher or lower than three times the standard deviation for outcome variables were identified, checked for possible unlikely values, and excluded in case of implausibility. To account for TEI, the residual method was chosen; TEI was regressed on total sugar calories and residuals were calculated. Those residuals were used in all following models and are referred to by naming sugar intake in the current analysis. The base model included country, sex, misreporting, and calories from total sugar. The following covariates were included stepwise into the base model and were tested for improvement of the model fit by improving adjusted *R*^2^, square root of the variance of the residuals, having normally distributed residuals, and whether the respective covariate reached significance: zBMI of the child, education of parents, smoking and alcohol drinking during pregnancy, maternal pre-pregnancy BMI, and physical activity. Furthermore, total sugar intake in the final model was exchanged for intake by major food groups of total sugar, including total sugar from milk products, fruit products (including fruit juice), confectionary, SSBs, and bread and cereals. Total sugar from those food groups was also included as residuals into the model.

For sensitivity analyses, analyses were repeated with crude blood marker concentrations, which were not standardized. All analyses were conducted in SAS 9.4 and a *p* value of ≤ 0.05 was selected as significance threshold.

## Results

Valid blood measurements were available for 438 children and nutrition data for 446 children at 8 years of age; both were available in 336 children. Eleven children were excluded due to implausible values in blood data. Thus, the analyzed data set consisted of 325 children from 5 different European countries, with the majority being from Spain and Italy (Table 1). Most parents had a high or medium level of education. Mean TEI at 8 years of age was 1587 (SD 278) kcal, with 332 (SD 110) kcal contributed from total sugar (21 energy % from total sugar). SSBs were consumed by 128 children (38%), whereas all other major total sugar-containing food products were consumed by most of the children. Most calories from total sugar were consumed as fruit products [mean intake 97 (SD 62) kcal/day; consumed by *n* = 276] and confectionaries [84 (SD 54) kcal/day; *n* = 285], followed by milk products [64 (SD 41) kcal/day; *n* = 287], SSBs [54 (SD 53) kcal/day; *n* = 128], and bread and cereals [18 (SD 21) kcal/day; *n* = 289].

**Table 1:** Baseline characteristics for study participants with nutritional information and blood measurements at 8 years of age

Characteristics^a^	Analysis cohort (*N* = 325)
Age (years)	8.0 (0.1)
Male	160 (49%)
Country	
Germany	24 (7%)
Belgium	35 (11%)
Italy	86 (26%)
Poland	71 (22%)
Spain	109 (34%)
Education of parents	
Low	26 (8%)
Middle	171 (53%)
High	127 (39%)
BMI	17.0 (2.8)
Dietary intake	
Energy intake (kcal/day)	1587 (278)
Energy percentage from fat	36 (9)
Energy percentage from protein	13 (4)
Energy percentage from carbohydrates	51 (9)
Total sugar (kcal/day)	332 (110)
Energy percentage from total sugar	21 (6)
Total sugar intake^b^	
Milk products (kcal/day)	64 (41)
Fruit products (kcal/day)	97 (62)
Sweetened beverages (kcal/day)	54 (53)
Bread and cereals (kcal/day)	18 (21)
Confectionary (kcal/day)	84 (54)
Blood marker^c^	
Glucose (mg/dl)	83.3 (8.4)
Insulin (µIU/ml)	9.3 (5.7)
HOMA index	1.8 (0.7)
TG (mg/dl)	64.5 (40.2)
TG (mmol/l)	0.74 (0.46)
HDL-C (mg/dl)	59.6 (15.1)
HDL-C (mmol/l)	1.55 (0.39)
TG/HDL ratio	1.1 (0.7)
TC (mg/dl)	167.5 (27.5)
TC (mmol/l)	4.35 (0.71)
LDL-C [mg/dl]	94.7 (25.0)
LDL-C (mmol/l)	2.46 (0.65)

*HOMA* High Homeostasis Model Assessment, *TG* triglycerides, *HDL-C* high-density lipoprotein cholesterol, *TC* total cholesterol, *LDL-C* low-density lipoprotein cholesterol

^a^Categorical variables are displayed as *N* (%) and continuous as mean (SD)

^b^Some food groups were not consumed by all children: milk products (*n* = 38), fruit products (*n* = 49), sweetened beverages (*n* = 197), bread and cereals (*n* = 36), confectionary (*n* = 40)

^c^Missings: insulin (11), HOMA index (35), triglycerides (1), HDL-C (1), TG/HDL ratio (1), LDL-C (4)

Mean serum concentrations of blood lipids and glucose-related blood markers are displayed in Table 1. There were differences between countries, with the highest mean concentrations of glucose and insulin in Poland (88 mg/dl) and Germany (15 µIU/ml), respectively, and lowest concentrations of both markers in Italy (glucose: 78 mg/dl, insulin 7 µIU/ml) (Supplementary Table 1). More favorable blood lipids (lower TG, TC, and LDL-C, and higher HDL-C concentrations) were generally seen in Italy and Spain and more unfavorable levels in Germany and Poland. Mean HDL-C concentrations ranged from 50 mg/dl (SD 11 mg/dl) in Poland to 64 mg/dl (SD 14 mg/dl) in Spain. Variations of blood values differed across countries. This supports the analysis of country-specific *z* scores. Blood marker concentrations were similar in both sexes. We also found BMI differences between countries, with the highest mean BMI in Spain (17.3 kg/m^2^) and Italy (17.5 kg/m^2^), and the lowest in Belgium (15.8 kg/m^2^). This was partly in line with caloric intake, which was reported highest in Spain and Poland and lowest in Belgium. Concerning total sugar intake, most total sugar was consumed in Poland (23 energy % from total sugar) and least of all in Italy (18 energy % from total sugar). TEI was higher in boys compared to girls, but energy percentage from total sugar intake did not differ between sexes. Sugar intake from milk products was highest in Spain, while sugar intake from fruit products, bread and cereals, and confectionary was consumed mostly in Germany. Highest sugar intake from SSBs was observed in Belgium. Sugar intake from major food products was very similar in boys and girls.

**Fig. 1:**
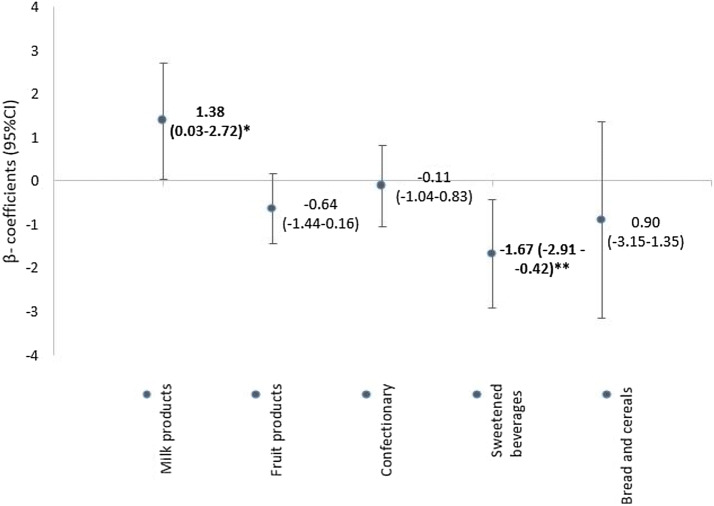
Linear regression coefficients for sugar intake from major food groups and their association with *z* score HDL levels^1^. All values are *β*-coefficients (95% CIs) for an increase of 100 kcal of sugar from specific food groups; *p = 0.045, **p = 0.009. Models were adjusted for sex, country, zBMI, and misreporting. ^1^HDL concentrations were included in the analysis after laboratory-specific standardization. *HDL*-*C* high-density lipoprotein cholesterol

Looking at the effects of total sugar intake and blood markers in the linear regression models, the covariates country, sex, zBMI, and misreporting showed either a significant improvement of model fit or confounding and were included in the final model. The fully adjusted models for each lipid marker or glucose-related marker are displayed in Table 2. An increase of total sugar intake by 25 g or 100 kcal per day was significantly associated with a lower HDL-C concentration *z* score (− 0.14; 95% CI − 0.01, − 0.27) which represents a decrease of 2.13 mg/dl (95% CI − 0.20, − 4.05 mg/dl), based on the mean and standard deviation across all countries. None of the other lipid markers or glucose-related markers was associated with total sugar intake. Similar results with slightly larger confidence intervals were seen in unadjusted models and in the sensitivity analysis using crude blood marker concentrations (Supplementary Table 2). Considering the five main different food groups for total sugar intake (total sugar from milk products, fruit products (including fruit juice), confectionary, SSBs, and bread and cereals), an increase by 100 kcal or 25 g/day of total sugar intake from SSBs was significantly negatively associated with HDL-C *z* score (− 1.67; 95% CI − 0.42, − 2.91), while total sugar intake from milk products was borderline, positively related to HDL-C *z* scores (1.38; 95% CI 0.03, 2.72) (Fig. 1). Similar results were observed in unadjusted models: the negative association of sugar from SSBs with HDL-C stayed significant, but sugar intake from milk products was not significant (Supplementary Table 3).

**Table 2:** Linear regression coefficients for total sugar intake (per 100 kcal) and their association with markers of sugar metabolism and lipids

	*β*-coefficient of *z* score	95% CI	*p* value
Sugar metabolism			
Glucose	0.03	(− 0.10 to 0.16)	0.652
Insulin	− 0.00	(− 0.11 to 0.11)	0.980
HOMA index	− 0.02	(− 0.15 to 0.11)	0.770
Blood lipids			
TG	0.08	(− 0.03 to 0.19)	0.168
HDL-C*	− 0.14	(− 0.27 to (− 0.01))	0.031
TG/HDL-C-ratio	0.02	(− 0.10 to 0.14)	0.774
TC	− 0.10	(− 0.23 to 0.04)	0.155
LDL-C	− 0.03	(− 0.17 to 0.10)	0.624

All values are *β*-coefficients (95% CIs) for an increase of 100 kcal of total sugar

Separated models of markers of sugar metabolism and lipids were each adjusted for sex, country, zBMI, and misreporting. Outcome variables were included in the analysis as *z* scores by laboratory

*TG* triglycerides, *HDL-C* high-density lipoprotein cholesterol, *TC* total cholesterol, *LDL-C* low-density lipoprotein cholesterol

^*^Significant

## Discussion

In the multicentric CHOP study, we observed a negative association of total sugar intake and total sugar intake from SSB with HDL-C blood concentrations in 8-year-old children. Other blood lipids and blood sugars were not significantly affected by total sugar intake. A major contributor for the negative association between total sugar intake and HDL-C levels was total sugar intake from SSBs. Total sugar intake from milk products was borderline, positively associated with HDL-C levels, which may reflect the close association of milk sugar and milk fat intake, with the latter being suggested to increase HDL-C [37–40].

The association of increasing sugar intake with decreasing HDL-C concentrations has been previously reported. Lee et al. observed a 0.26 mg/dl greater increase in HDL-C levels, comparing lower (< 10% of TEI) to higher added sugar consumption (> 10% of TEI) in 2379 girls aged 9 and 10 years [19]. In two other studies comparing added sugar consumption below 10% of TEI to one above 22.8% [22] and 30% [17], a decrease of 3.1 mg/dl and 4.5 mg/dl HDL-C concentration, respectively, was observed in a high added sugar diet in 4047 (12–19 years) and 2252 (13–18 years) adolescents. This is comparable to our observed HDL-C decrease of 2.13 mg/dl with a 100 kcal higher intake of total sugar. Furthermore, decreased HDL-C concentrations with increasing SSB consumption was also observed by other studies [20, 21]. In contrast, the increased HDL-C level seen with higher total sugar intake from milk products in our study is more likely related to the higher milk consumption and not to the sugar, as suggested by several other studies [37–39]. Furthermore, the association of sugar from milk products with HDL-C has to be interpreted with caution, as it was only borderline significant and reached no significance in the unadjusted model. Additionally, it has to be kept in mind that SBBs were consumed only by 128 children compared to 287 children consuming milk products.

No significant associations were observed between total sugar intake and TG, TC, TG/HDL-C ratio, or LDL-C levels in the current study. Whereas this was also seen in one other study [20], most other studies found significant relations between sugar intake and blood lipids other than HDL-C in children and adolescents [17, 18, 21, 22, 41]. TG were especially positively associated with increased sugar intake in a number of studies [17–19, 21, 22]. Additionally, increased TG/HDL-C ratio [17] and LDL-C [41] have been observed during increased sugar intake. A meta-analysis of randomized controlled trials by Te Morgenga et al. concluded that higher compared with lower sugar intakes were significantly associated with increased TG, TC, and LDL-C concentrations in adults [16].

We did not see a direct association of total sugar intake with fasting blood sugar levels. An association of sugar intake with glucose, insulin, and HOMA levels has been inconsistently reported [17, 23–26, 42, 43]. Overall epidemiological evidence on the effect of sugar intake on blood glucose metabolism is limited, and the current evidence on the influence of sugar intake on the development of diabetes, obesity, or CVD is inconclusive [26–28, 42, 44].

Possible causes for inconsistencies across studies might be due to the different types of sugars assessed across studies. While some studies investigated mainly total or added sugars, others focused on specific type of sugars such as fructose. Fructose is related to fat metabolism and might influence blood sugars and lipids in a different way as total sugars, which includes also other mono- and disaccharides [27, 45, 46]. Furthermore, different approaches to assess sugar intakes from different food groups might also contribute to inconsistent results across studies, along with the close association of lactose intake with the intake of other nutrients from dairy products. It was proposed that the effects of sugar intake may also differ depending on the food matrix and whether it is taken in solid or liquid form [24, 27].

Sugar intake in the CHOP trial is comparable to intake in other European countries [13, 31, 47]. Although sugar intake in Europe seems to plateau and even to decrease in some parts in children and in adults [13, 14], high sugar intake and free sugar intake above the WHO recommendations of 10% are widely prevalent [12]. Besides the potential unfavorable influence of increasing sugar intake on HDL-C concentrations, high sugar intake is suggested to promote the development of dental caries, overweight and obesity, and the metabolic syndrome [12]. Therefore, useful interventions and recommendations to reduce sugar intake are needed to prevent potential detrimental effects caused by a high sugar consumption.

## Strengths and limitations

One strength of our study is accurate nutritional assessment, anthropometric measurements, and blood draw using the same standard operating procedures across study sites by jointly trained study nurses. We could explore effects of diet across five European countries. The technique used for dietary assessment is a major advantage of our study. Dietary records on three consecutive days were used, which are considered as the most precise method of dietary intake assessment in children for large studies [30].

One limitation is that most blood lipid and sugar concentrations were measured in different local laboratories, which may have added imprecision. Additionally, venous blood in Germany was mostly drawn in the afternoon without an at least fasting period of 6 h. To reduce the potential bias and measurement error, we calculated laboratory-specific standardized values. We did not separate natural and added sugars due to the chosen dietary assessment and nutrition database. Therefore, comparison of our results to those studies that focused on added sugars is challenging. Although 3-day weighed dietary records are considered as the most precise method of dietary intake assessment, correct estimation of sugar intake is still limited by self-reported data [48] and results have to be interpreted with caution. Furthermore, SSBs were consumed by less than half of the children, which has to be kept in mind while interpreting the results. Generalizability of the study result might be limited since predominantly families with middle and high education participated in the follow-up.

## Conclusion

In a cross-sectional multicentic study in 8-year-old European children, increasing total sugar intake, especially from SSBs, was associated with an unfavorable decrease of HDL-C levels. The association between dietary sugar intake during childhood, particularly from SSBs, and long-term cardiovascular risk warrants further research.

## Electronic supplementary material

Below is the link to the electronic supplementary material.

Supplementary file1 (PDF 83 kb)

Supplementary file2 (PDF 195 kb)

Supplementary file3 (PDF 153 kb)

## Abbreviations

BMI: Body mass index
CHOP: Childhood Obesity Project
CVD: Cardiovascular disease
TEI: Total energy intake
HDL-C: High-density lipoprotein cholesterol
LDL-C: Low-density lipoprotein cholesterol
NCD: Non-communicable disease
SSB: Sugar-sweetened beverage
TC: Total cholesterol
TG: Triglycerides
WHO: World Health Organization
