# Supplementary material for: Influence of total sugar intake on metabolic blood markers at 8 years of age in the Childhood Obesity Project
Source: Eur J Nutr. 2020 May 6;60(1):435–42. doi: 10.1007/s00394-020-02229-w (PMC7867537; doi:10.1007/s00394-020-02229-w)
Supplement: Supplementary file 1 — Supplementary file1 (PDF 83 kb) [file 394_2020_2229_MOESM1_ESM.pdf]

Supplementary Table 1: Baseline characteristics for study-participants with nutritional information and blood measurements at 8 years of age per country and gender

**Characteristics**[1]	**Country**					**Gender**	
	Germany (N=24)	Belgium (N=35)	Italy (N=86)	Poland (N=71)	Spain (N=109)	Male (N=160)	Female (N=165)
**BMI**	16 (2)	16 (2)	18 (3)	17 (3)	17 (3)	17 (3)	17 (3)
Total energy intake (kcal)	1642 (329)	1410 (263)	1511 (260)	1648 (168)	1650 (307)	1653 (277)	1522 (265)
Total sugar intake (kcal)	398 (167)	332 (119)	277 (91)	381 (93)	328 (93)	343 (117)	321 (102)
Energy percentage from total sugar	24 (7)	23 (6)	18 (5)	23 (5)	20 (5)	21 (6)	21 (6)
**Total sugar intake**[2]							
Milk products (kcal)	58 (27)	36 (29)	33 (22)	61 (36)	90 (41)	65 (42)	62 (41)
Fruit products (kcal)	148 (95)	75 (62)	85 (47)	120 (63)	81 (50)	104 (68)	90 (56)
Sweetened beverages (kcal)	42 (28)	104 (136)	49 (41)	50 (43)	61 (60)	54 (49)	54 (57)
Bread and cereals (kcal)	32 (35)	15 (14)	9 (12)	24 (26)	16 (15)	19 (25)	17 (17)
Confectionary (kcal)	108 (54)	104 (62)	61 (37)	105 (62)	73 (47)	86 (61)	82 (46)
**Blood marker**[3]							
Glucose [mg/dl]	87.5 (12.1)	86.3 (5.4)	78.3 (8.4)	88.5 (5.9)	81.9 (6.8)	84.6 (8.3)	82.1 (8.3)
Insulin [µIU/ml]	14.6 (17.2)	8.4 (2.2)	7.3 (3.0)	9.1 (3.0)	10.0 (3.4)	9.4 (7.4)	9.1 (3.4)
HOMA index	3.6 (5.7)	1.8 (0.6)	1.4 (0.6)	2.0 (0.7)	2.0 (0.8)	1.8 (0.7)	1.9 (0.8)
TG [mg/dl]	129.6 (96.8)	60.3 (27.1)	59.8 (22.2)	73.2 (31.9)	49.4 (18.1)	64.3 (47.6)	64.7 (31.5)
HDL-C [mg/dl]	57.7 (12.1)	63.6 (16.9)	61.2 (16.0)	50.4 (11.5)	63.5 (14.1)	61.2 (16.1)	58.1 (13.9)
TG/HDL-C ratio	2.6 (2.4)	1.1 (0.8)	1.1 (0.6)	1.6 (0.9)	0.8 (0.4)	1.0 (0.6)	1.2 (0.8)
TC [mg/dl]	170.4 (24.1)	167.8 (22.4)	165.2 (30.7)	167.6 (30.5)	168.5 (25.3)	166.4 (27.3)	168.5 (27.8)
LDL-C [mg/dl]	90.4 (26.4)	92.7 (23.1)	89.0 (24.7)	102.9 (26.9)	95.3 (23.0)	91.6 (24.8)	97.7 (24.9)

[1]*Categorical variables are displayed as N (%) and continuous as mean (SD).*
[2] *Some food groups were not consumed by all children: milk products (n= 38), fruit products (n= 49), sweetened beverages (n= 197), bread and cereals (n= 36), confectionary (n= 40).*
[3] *Missings: Insulin (11), HOMA index (35), Triglycerides (1), HDL-C (1), TG/HDL-C ratio (1), LDL-C (4).*
Abbreviations: High homeostasis model assessment (HOMA), Triglycerides (TG), High density lipoprotein cholesterol (HDL-C), Total cholesterol (TC), Low density lipoprotein cholesterol (LDL-C)
