# Supplementary material for: Influence of total sugar intake on metabolic blood markers at 8 years of age in the Childhood Obesity Project
Source: Eur J Nutr. 2020 May 6;60(1):435–42. doi: 10.1007/s00394-020-02229-w (PMC7867537; doi:10.1007/s00394-020-02229-w)
Supplement: Supplementary file 2 — Supplementary file2 (PDF 195 kb) [file 394_2020_2229_MOESM2_ESM.pdf]

Supplementary Table 2: Linear regression coefficients for total sugar intake (per 100kcal) and their association with markers of sugar metabolism and lipids in an unadjusted model and using crude blood marker values.

	Unadjusted model[1]			Crude values[2]		
	β-coefficient of z- score	95% CI	P-value	β-coefficient of crude values	95% CI	P-value
**Sugar metabolism**						
Glucose	0.03	(-0.10─0.15)	0.679	0.39	(-0.60─1.38)	0.438
Insulin	-0.25	(-0.13─0.08)	0.650	0.47	(-0.26─1.21)	0.207
HOMA-Index	-0.05	(-0.18─0.07)	0.389	0.02	(-0.07─0.11)	0.675
**Blood lipids**						
TG	0.07	(-0.03─0.17)	0.172	1.82	(-2.88─6.52)	0.447
HDL-C*	-0.12	(-0.24─ (-0.00))	0.049	-2.47	(-4.32─ (-0.61))	0.009
TG/HDL-C-ratio	0.01	(-0.10─0.12)	0.852	0.09	(-0.00─0.19)	0.054
TC	-0.08	(-0.20─0.05)	0.219	-2.87	(-6.58─0.83)	0.128
LDL-C	-0.03	(-0.15─0.10)	0.672	-0.82	(-4.09─2.45)	0.622

Note: All values are β-coefficients (95% CIs) for an increase of 100kcal of total sugar;
[1]Separated unadjusted models of markers of sugar metabolism and lipids. Outcome variables were included in the analysis as z scores by laboratory.
[2]Separated models of markers of sugar metabolism and lipids were each adjusted for sex, country, zBMI, and misreporting. Outcome variables were included in the analysis as crude values.
**=significant.*
Abbreviations: Triglycerides (TG), High density lipoprotein cholesterol (HDL-C), Total cholesterol (TC), Low density lipoprotein cholesterol (LDL-C)
