# Supplementary material for: Influence of total sugar intake on metabolic blood markers at 8 years of age in the Childhood Obesity Project
Source: Eur J Nutr. 2020 May 6;60(1):435–42. doi: 10.1007/s00394-020-02229-w (PMC7867537; doi:10.1007/s00394-020-02229-w)
Supplement: Supplementary file 3 — Supplementary file3 (PDF 153 kb) [file 394_2020_2229_MOESM3_ESM.pdf]

Supplementary Table 3: Unadjusted linear regression coefficients for sugar intake from major food groups and their association with z- score HDL-C levels[1]

Total sugar intake from	β-coefficient of z- score	95% CI	P-value
Milk products	0.98	(-0.18─2.14)	0.098
Fruit products	-0.51	(-1.26─0.25)	0.190
Sweetened beverages	-1.86	(-3.08─0.65)	0.003
Bread and cereals	-0.63	(-2.79─1.54)	0.570
Confectionary	-0.17	(-1.05─0.71)	0.705

Note: All values are β-coefficients (95% CIs) for an increase of 100kcal of sugar from specific food groups;
[1]HDL-C concentrations were included in the analysis after laboratory specific standardization.
Abbreviation: High density lipoprotein cholesterol (HDL-C)
